# Ventricular tachycardia after administration of sildenafil citrate: a case report

**DOI:** 10.1186/1752-1947-1-65

**Published:** 2007-08-13

**Authors:** Jeppe G Rasmussen, Egon Toft, Ole Frøbert

**Affiliations:** 1 Department of Cardiology, Center for Cardiovascular Research, Aalborg Hospital, Aarhus University Hospital, Hobrovej 18-22, 9000 Aalborg, Denmark; 2 Department of Health Science and Technology, Center for Sensory-Motor Interaction, University of Aalborg, Fredrik Bajers Vej 7 D3, 9220 Aalborg, Denmark; 3 Department of Pharmacology, University of Aarhus, Bartholin Bygningen, 8000 Aarhus, Denmark

## Abstract

**Background:**

It has not previously been reported that sildenafil citrate causes malignant arrhythmias in humans.

**Case presentation:**

A 41-year-old man developed sustained ventricular tachycardia following sildenafil citrate administration.

**Conclusion:**

It cannot be dismissed that this patient experienced ventricular tachycardia as an adverse effect of sildenafil citrate administration.

## Background

Concerns about the safety of sildenafil citrate (Viagra^®^) have previously been raised [1]. Other case reports and studies have described electrophysiological changes associated with ventricular arrhythmias following administration of sildenafil citrate [2-4].

We describe a case of a young man with no cardiac history or family history of heart disease or sudden death, developing sustained ventricular tachycardia after ingestion of sildenafil citrate. This case highlights a potential adverse effect of sildenafil citrate and the possible morbidity and potential lethality associated with this adverse effect.

## Case presentation

**Figure 1:**
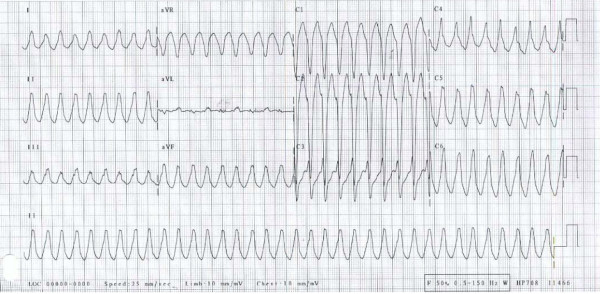
Sustained ventricular tachycardia in a 41-year-old man receiving sildenafil therapy. Original tracing when the patient was admitted to the hospital.

In August, 2006, a 41-year-old man was transferred after an episode of sustained monomorphic ventricular tachycardia (VT). There was no history of any cardiopulmonary symptoms and no family history of heart disease or sudden death. The arrhythmia started after 90 minutes of moderate pace swimming. In the locker room the patient felt dizzy, had a feeling of tachycardia and experienced a brief, witnessed syncope. At admission to the referring hospital VT with a frequency of 220 min^-1 ^was documented (figure 1). Blood pressure was 105/60 mmHg. Before transferral, the VT was treated with metoprolol 2 milligrams and amiodarone 300 milligrams intravenously and converted to sinus rhythm.

**Figure 2:**
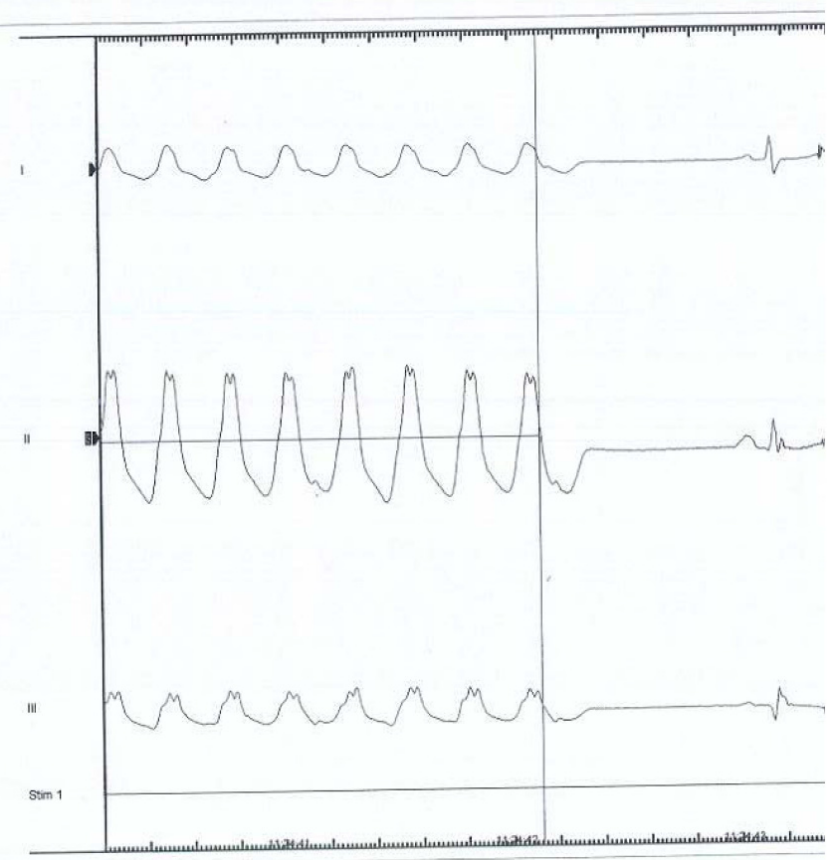
Ventricular tachycardia induced during electrophysiological study. The study was conducted 41/2 hours following administration of 100 mg of Viagra^® ^orally. A non-sustained VT over 24 beats identical to the VT at admission was induced.

Approximately 10 hours prior to the arrhythmia the patient had taken sildenafil citrate (Viagra^®^) 100 mg orally, which had been prescribed because of erectile dysfunction. He was not taking any other medication. Blood tests were unremarkable except for troponin T (0.82 mikrograms/Liter) and CKMB (40.8 mikrograms/Liter). Resting ECG, bicycle ergometer exercise testing, echocardiography with tissue Doppler imaging, coronary angiography and cardiac nuclear magnetic resonance scanning were all normal. Six endomyocardial biopsies from the right ventricle showed slight non-specific hypertrophy and slight interstitial fibrosis and no suspicion of arrhythmogenic right ventricle cardiomyopathy. An electrophysiological study using protocol stimulation in the apex and the outflow tract of the right ventricle induced 10 beats of non-sustained VT with alternating morphology. On a subsequent day, an additional electrophysiological study was conducted 41/2 hours following administration of 100 mg of Viagra^® ^orally (serum concentration 0.36 mg/kg). On that occasion a non-sustained VT over 24 beats identical to the VT at admission was induced (figure 2). A prophylactic implantable cardioverter defibrillator (ICD) was implanted and the patient was instructed not to use sildenafil. At a follow-up visit in December 2006 interrogation of the ICD revealed that two events of VT had been detected and terminated by anti tachycardia pacing.

## Discussion and conclusion

Sildenafil citrate is widely used to treat male erectile dysfunction and therapeutic uses for other diseases such as pulmonary hypertension are emerging. Sildenafil citrate has proven to be safe when used in the treatment of erectile dysfunction of organic, psychogenic or mixed causes [5]. Concerns about the effects of phosphodiesterase-5 inhibitors on the heart and their safety in patients with cardiovascular disease have been raised. The concerns include effects on blood pressure, heart rate and cardiac electrophysiology. This led to the publishing of a report from the U.S. Food and Drug Administration, describing deaths in patients having been prescribed sildenafil citrate in the first period after the marketing from late March through mid-November 1998 [1]. In this period, >6 million outpatient prescriptions were dispensed. Of the 130 confirmed deaths among men (mean age, 64 years) who received sildenafil citrate, 77 had cardiovascular events, including 41 with myocardial infarction and 27 with cardiac arrest. Cause of death was unknown in 48 and non-cardiac in 5 men. The time from drug ingestion to death or onset of symptoms leading to death was <5 hours for 44 men, later the same day; 6 men, the next day; 8 men, two to seven days later; 9 men and unknown for the remainder. Sildenafil citrate blocks the rapid component of the delayed rectifier potassium current in guinea pig hearts [2] and produces small but significant increases in the QTc interval in humans [3]. Sildenafil has been reported to cause VT in pigs when administered in combination with a nitric oxide donor [4].

We conclude that our patient had no confirmable cardiac condition other than VT. The hypothesis, that this patient, with documented episodes of non-sustained VT, experienced an episode of sustained VT caused by sildenafil citrate lowering the VT threshold, cannot be dismissed.

## Abbreviations

1. VT: Ventricular tachycardia

2. ICD: Implantable cardioverter defibrillator

## Competing interests

The author(s) declare that they have no competing interests.

## Authors' contributions

All authors were involved in writing/reviewing the manuscript. All authors approved the final manuscript.
